# Inheritance and Innovation of Pottery Sculpture Technique in Shiwan, China: A Grounded Study from Cultural Ecology

**DOI:** 10.3390/ijerph20043344

**Published:** 2023-02-14

**Authors:** Liting Zhou, Xiaomei Zou, Yongnan Huang, Yiyong Li, Luyao Guo, Junyu Fu

**Affiliations:** 1 School of Urban Culture, South China Normal University, Guangzhou 510631, China; 2 School of Educational Information Technology, South China Normal University, Guangzhou 510631, China; 3 School of International Business, South China Normal University, Guangzhou 510631, China

**Keywords:** ecological innovation, social participation, environmental protection, grounded theory, Shiwan pottery sculpture technique

## Abstract

Ceramics play an important role in human daily life and production practice. Pottery sculpture technique is the core of ceramic making. However, the production process of traditional ceramics is accompanied by high pollution, which has a great impact on human health and the ecological environment. Rapid development of industrialization has exacerbated this consequence. As the “Pottery Capital of Southern China”, Foshan has been involved in environmental crises while relying on the ceramic industry to develop. Since the 21st century, Foshan has gradually successfully driven to upgrade the city from industrial to culture-led by carrying out positive innovations in Shiwan pottery sculpture technique. Therefore, based on the theoretical perspective of cultural ecology, this paper selects Shiwan pottery sculpture technique as the object, uses Python (Octopus Collector) to obtain data, and applies grounded theory to generate the ecological evolution model. This study discussed how the Shiwan pottery sculpture technique promotes the harmonious coexistence of human beings, industries, and cities in the new cultural ecological environment of the 21st century by exploring and clarifying the interaction and function of different elements in different stages of evolution. Finally, this study not only makes up for the current lack of research on Shiwan’s cultural ecology, but also provides meaningful reference for environmental reform in other industrialized cities.

## 1. Introduction

In the past few decades, industrial production has accelerated the economic development of various countries, making continuous improvements in people’s material living standards. However, people paid too much attention to the current economic interests and neglected the sustainable development of the environment. As a result, excessive industrialization caused great damage to the ecology, affected the carrying capacity of the natural environment, and caused resource depletion, severe environmental pollution, unhealthy lifestyles of human beings, and other issues. Later, the countries gradually realized this problem and began to seek reasonable solutions. For example, adjusting the industrial structure, formulating stricter environmental protection standards, and emphasizing that the companies should use green technology in the production processes as much as possible. According to the study, the quality of the environment directly affects human health and acts on all aspects of production and life, ultimately having an impact on future human well-being [1]. The Millennium Ecosystem Assessment Report organized by the United Nations in 2005 pointed out that ecosystem services are closely related to human well-being [2]. Therefore, paying attention to the environmental ecosystem can help us regulate the overall layout of cities in a more scientific way.

During an eating activity after an occasionally rainy day, primitive humans unexpectedly found that fire can harden wet clay, which eventually led to the birth of later pottery. Subsequently, ancestors cleverly used the clay and made it a ceramic vessel through technical improvements [3]. Thanks to the huge functional value that ceramics brought to human life, nowadays ceramics have developed into products that can be seen everywhere in daily life. Industrial products produced by ceramic sculpture techniques have been widely used in public and family life, while artistic products have been used to improve the aesthetic taste and spiritual realm of humans, playing an important role in the realization of human well-being. However, the materials used in the production process of traditional ceramics, such as soil and pigments, contain a large number of heavy metals, which have great damage to human health and ecosystems such as land, dust and wastewater pollution [4], human respiratory diseases [5], cardiovascular and cerebrovascular diseases [6], and other health risks. China is the ancient ceramic capital with a long history of exquisite workmanship; as a country with the largest production of traditional ceramics in the world today, the ceramic industry has already occupied a pivotal position in China’s national economy. For example, in 2019, the ceramic industry accounted for about 7.06% of the gross domestic industrial production [7,8], becoming the economic pillar industry of many cities. Foshan, Guangdong has a well-developed pottery industry and a long history of pottery culture. It is reputed as the “Pottery Capital of Southern China” and is also the largest production base of architectural and sanitary ceramics in contemporary China. Its pottery resources are mainly concentrated in Shiwan town of the Chancheng District. The phrase “Shiwan Ridge Tiles, the best in the world” points out the important position of Shiwan ceramics in the history of Chinese ceramics [9]. To vigorously develop the economy, Foshan’s ceramic industry in history produced a large number of daily-use ceramics, which caused great damage to the environment and human health. Since the 21st century, to promote the transformation and upgrading of Foshan City and seek a more long-term sustainable development model, the local government has set the goal of building a culturally oriented city, taking the Shiwan pottery sculpture technique as a significant support object and making positive and beneficial innovations to it [10]. These measures are not only to improve the production technology, thus reducing the environmental and human hazards, but also to emphasize its cultural attributes and reconstruct the economic development model by linking various industrial forms. At the same time, ceramics are also widely used in urban public spaces because of their unique material characteristics and artistic expression [11]. In a word, based on the current environmental protection background and goal, Foshan provided diversified values for various elements of the ecology through the inheritance and innovation of the Shiwan pottery sculpture technique. Finally, Foshan explored a more sustainable development model to meet the requirements of citizens for a better life.

The development model of Shiwan can provide case guidance for the transformation and upgrading of other cities to a certain extent. In order to better explore this process, the paper introduces the theory of cultural ecology. Cultural ecology is an interdisciplinary theory, which was first proposed by Julian Steward in 1955 [12]. In this theory, he put forward the idea of “multi-linear evolution”, and made a detailed analysis based on the clue of “cultural ecology”, emphasizing that culture and the ecological environment cannot be separated, and will have mutual influence or effect. Later, after continuous improvement, and with the constant expansion of the cultural category, it was widely used in biology, philosophy, geography, pedagogy, and other fields. For example, Marvin Harris proposed “cultural materialism” [13], Carl Ortwin Sauer studied the interrelationship between cultural landscape and the ecological environment [14], and Yunjie Sima improved the multilayer composition of cultural ecology [15]. In practical application, the research on specific cases based on the theoretical framework accounts for a considerable part, and also conducted a few discussions like Colleen Chiu Shea’s on how to carry out urban planning and institutional design for several demonstrative eco cities in China [16]. Alexandra S. Wormley et al. established an eco-cultural dataset to facilitate the comparison of different cultural ecology [17], etc. In general, although there have been many studies on cultural ecology, only a small part of them involve the extraction and presentation of models [18,19,20,21]. On the other hand, the current investigation on the relationship among city, ecology, and culture is still relatively fragmented [22]. Therefore, it is necessary to re-investigate the cultural ecology that is closely related to the city in the way of model construction. In addition, the state of culture can be measured more effectively through cultural ecology.

Based on the above discussion, this paper selects the Shiwan pottery sculpture technique as the object of study, and deeply explores how it has formed a new cultural ecology under the background of the era of Foshan vigorously emphasizing environmental protection. Through the application of grounded theory, the study analyzed about 860,000 Chinese language texts, establishing the cultural ecological evolution model of Shiwan pottery sculpture technique. This model not only divides and explains the reasons for the changes of cultural ecology in different stages and identifies the inheritance and innovation path of Shiwan pottery and plastic technology, but also expounds in detail the roles of different elements such as policy objectives, social participation, technological innovation, cross-regional communication, etc., and reveals the function and significance of the new cultural ecology.

## 2. Materials and Methods

### 2.1. Research Background

This section aims to help readers better understand the development process of the Shiwan pottery sculpture technique by successively discussing the natural environmental conditions and the role of social factors in different periods in the past. Moreover, the research describes the technological process of the Shiwan pottery sculpture technique, showing the artistic characteristics of this culture and the impact of its production process on the environment. These contents laid the foundation for later research.

#### 2.1.1. Forming Conditions

**Figure 1:**
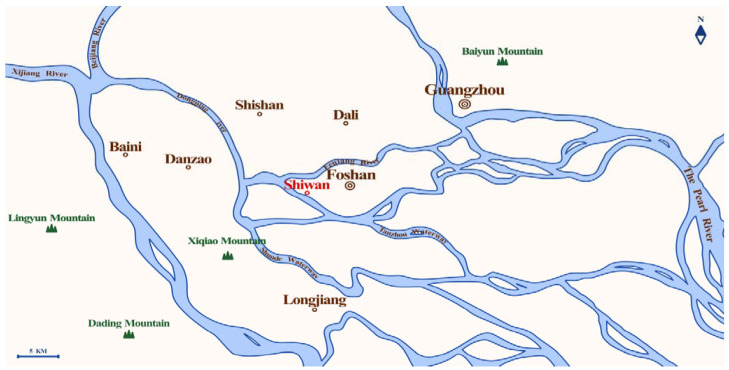
Geographical location of Shiwan.

**Figure 2:**
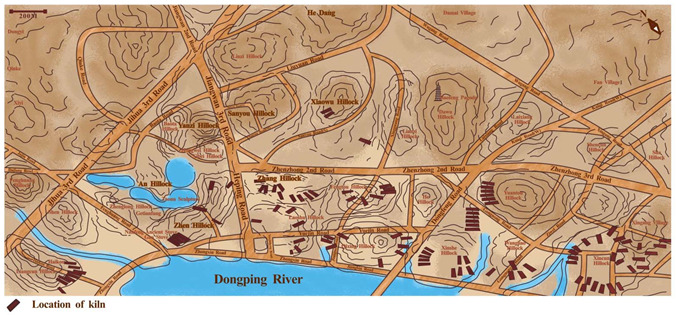
The location of ancient Shiwan kilns and hillocks on the modern map.

As a result of environmental adaptation, the culture of Shiwan pottery sculpture technique cannot be separated from the local unique geographical location, natural resources, climatic conditions, and so on. Shiwan was originally under the jurisdiction of Shizhou Village, Foshan Town, Nanhai County; the geographical location of Shiwan kiln can be divided into a narrow and broad sense [23] (p. 9). The narrow sense of Shiwan kiln usually refers to the north and northeast banks of Dongping River in Foshan, the narrow strip west of Dawugang, east of Haikou, and north of Lanshi (Figure 1). There are many rolling hills distributed in this area, most of which are red earth hills formed in the Quaternary period. The soil quality is excellent, and it contains rich clay and sandy soil, providing high-quality fetal soil for pottery making. More than 100 dragon kilns were built in succession on the hillocks near the river and where the potters gathered in the town [24]. The branches of Dongping River are densely distributed, providing an inexhaustible source of water and water transportation for pottery making. The metal-rich silt at the bottom of the river, shells on the river beach, mulberry trees and straw on the hills provide rich raw materials for glaze making [25] (pp. 73–77). At the same time, Shiwan’s land transportation is also very developed; especially the railways and highways provide great convenience for transportation of raw materials and products of ceramic industry [24]. The core area of modern Shiwan pottery and the ancient terrain has changed a lot, the ancient Jiangwan has now become a flat land [23] (p. 9) (Figure 2). In terms of climate conditions, Shiwan is located in the area of 113°10′ to 113°12′ east longitude and 23° to 23°2′ north latitude. The village is about 6 square kilometers. It is a subtropical area with a mild climate, abundant rainfall, and sufficient sunshine, like spring all the year round [23] (p. 14).

#### 2.1.2. Historical Traceability

The development of Shiwan pottery sculpture has a long history which can be traced back to the imprinted pottery at the Hedang Shell Mound Site in the late Neolithic [25] (p. 35). The Tang Dynasty was the initial development period of the Shiwan pottery industry. During this period, it mainly focused on the production of daily pottery with the main shapes of bowls, plates, basins, pots, jars, etc., as well as funeral supplies such as soul bottles and tripod stoves and so on. By the Song Dynasty, China’s commerce was highly developed, and the consumption fashion of the whole society promoted the unprecedented development of the ceramic industry. This period was the heyday of Shiwan ceramics. The kilns engaged in ceramic production spread all over the country. The varieties of daily-use ceramics, architectural, or garden ceramics and artistic ceramics increased dramatically, and their shapes and styles were increasingly renovated. With the expansion of foreign trade, the ceramic industry gradually developed from the mainland to coastal areas such as Zhejiang, Fujian, Guangdong, Guangxi, and other places. From the Southern Song Dynasty to Yuan Dynasty, a large number of immigrants from the Central Plains settled in Foshan. They brought the northern ceramic technology to Shiwan, and integrated it with original local pottery-making technology, greatly improving the manufacturing level and artistic standard of Shiwan pottery [25] (pp. 43–50). Since the Ming Dynasty, Shiwan has broken the previous situation of single exporting daily-use ceramics. Artistic pottery, architectural garden ceramics, handicraft pottery, and so on have also been continuously exported, especially the architectural garden ceramics which are widely welcomed by Southeast Asia. After the Ming Dynasty, the variety and themes of ceramics gradually became wider and wider, fully reflecting the characteristic of the civilian. During the Ming Jiajing period (1522–1566), pottery guilds appeared in Shiwan. In the middle of the Ming Dynasty, eight pottery guilds had been formed in Shiwan. By the Qianlong period of the Qing Dynasty, they had developed to 22 guilds, and by the end of the Qing Dynasty, they had stabilized at 24 guilds. There were thousands of varieties of pottery, not only for daily use but also for the production of artistic pottery sculptures and garden architectural pottery, which had a wide influence [25] (pp. 53–56).

After the collapse of the Qing government, Shiwan ceramics suffered a serious decline in the Republic of China era. By the early days of liberation, there were only a dozen kilns in the whole industry of Shiwan, of which only seven were in production, and there were only a thousand potters [25] (p. 64). After the founding of the People’s Republic of China, Shiwan pottery was reborn, gradually established cooperatives, followed by art ceramics factories. Moreover, the local government also invited countless folk pottery sculptors who had left their hometown or changed profession for a living back to Shiwan to continue their pottery creation. During this time, Shiwan pottery gained different degrees of reform and innovation from theme, shape, to technique, and the ceramic art level has been steadily improved [25] (pp. 65–66). During the Cultural Revolution, Shiwan pottery suffered severe damage and destruction. Many excellent arts and crafts treasures were destroyed. Many creators, artists, and technicians were persecuted, resulting in a sharp decline in production, varieties, and heavy losses of the whole industry. After the end of the ten-year catastrophe, especially the reform and opening up ushered in the spring of the arts and crafts industry, and since then Shiwan pottery has achieved unprecedented development and remarkable achievements. Quite a few works have been selected for exhibition in Hong Kong, Macao, America, England, Canada, and other countries and regions, winning the favor and high praise of people at home and abroad [24].

It can be seen from the historical development that, as a kind of folk kiln product, Shiwan pottery is produced for the needs of the market and the masses and goes to the whole world for the export needs. Countless people use their wisdom to create a rich variety of ceramics and bring positive changes to their lives.

#### 2.1.3. Technological Process

Shiwan pottery sculpture technique is a comprehensive process of mud, glaze, and fire, and is also a transcender that is based on fully absorbing the advantages of various famous kiln products [24]. It can be divided into four major procedures of raw material processing, adobe molding, glazing, and calcination in order.

Refining: Mud is the basic raw material for pottery forming; the traditional raw material processing is manually operated. In this process, the mud with different origin, different molding and firing performance is put in the mud tank and soaked in water, then picked up and repeatedly stirred and trampled until the mud is mature enough to meet the needs of the manufacturer. In order to improve the performance of clay, Shiwan artists will add clinker (such as tile powder) for different purposes during the production process [25] (pp. 89–91). Due to the presence of heavy metal elements in the raw material, some degree of contamination is usually accompanied in the subsequent calcination.

Modeling: The preparation of throwing is kneading mud. Handmade throwing is the longest and most productive molding method for Shiwan kiln products. The difference between manual throwing of Shiwan kiln and other porcelain areas is the use of stoneware, which is more malleable. In terms of molding technique, in addition to the usual methods of carving, faceting, engraving, and scratching, Shiwan ceramics also have four other methods of pasting, kneading, impressing, and engraving to enhance local expression and decoration [25] (pp. 94–99). In fact, in the process of shaping works, except for individual works, most of Shiwan pottery sculptures use several techniques together and penetrate each other.

Glazing: Glazing is the process of applying glaze slurry on the surface of the shaped ceramic body. Different kiln changes will appear with the different choice of glaze slurry and temperature in the process of calcination, which is actually a creative process. For a long time, the coloring agent used in Shiwan kiln is the silt in Dongping River, and the metal composition in the silt is mainly iron oxide. If the silt is mixed with straw ash, mulberry ash, and pine wood ash, it will become sauce yellow glaze and yellow-brown glaze [25] (pp. 103–105).

**Figure 3:**
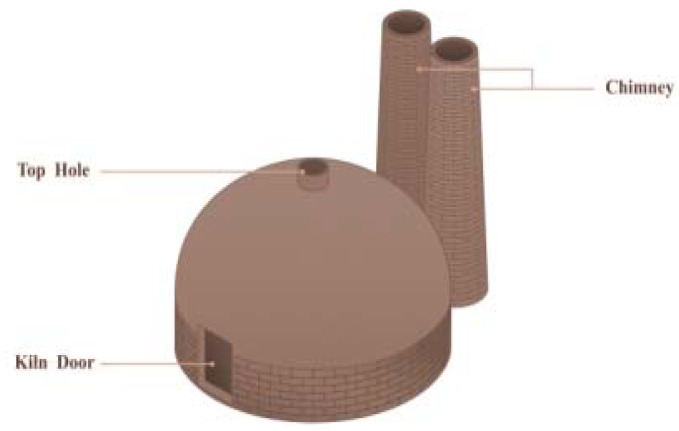
External view of Steamed Bun Kiln.

**Figure 4:**
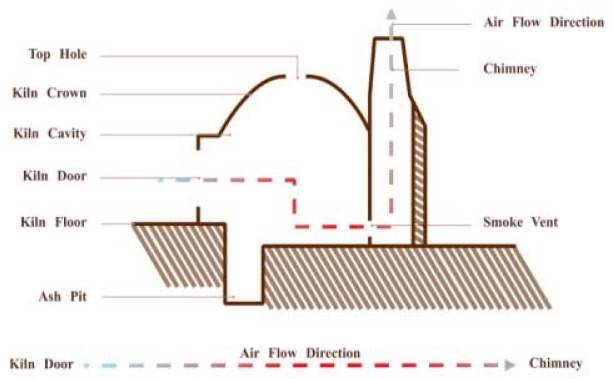
Internal configuration of Steamed Bun Kiln.

**Figure 5:**
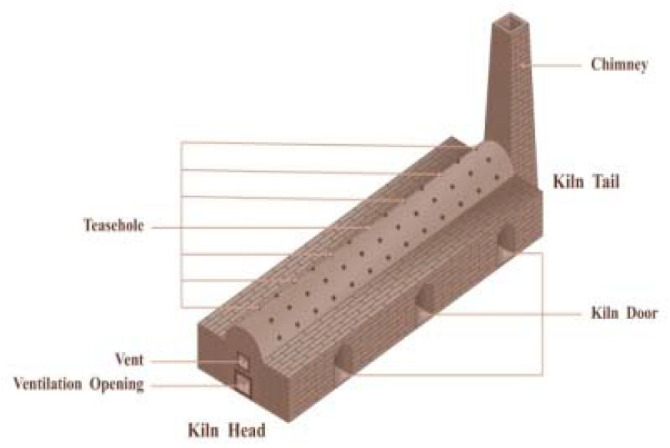
External View of Dragon Kiln.

**Figure 6:**
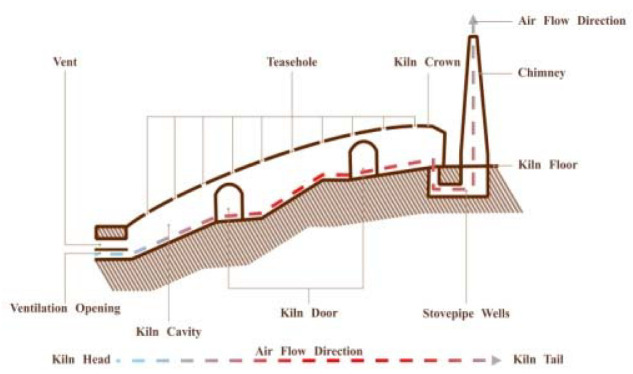
Internal Configuration of Dragon Kiln.

Firing: The calcination of Shiwan kiln is divided into several steps such as preheating, firing, and high-temperature reduction. According to the archaeological evidence, the earliest ancient kiln site discovered in Shiwan appeared in the Tang Dynasty, which is a semi-inverted flame steamed bun kiln site (Figure 3) [23] (p. 21). The firing chamber of this kiln is in front of the kiln bed, and the chimney is set at the back of the kiln. The flame sprays from the fire chamber to the kiln crown and then falls to the kiln bed, flows through the blank, and then the smoke is discharged out of the kiln through the smoke outlet and the vertical flue, and the flame is half inverted [26] (p. 378) (Figure 4). Later, it gradually becomes a “Dragon Kiln” leaning upwards against the mountainside (Figure 5). Shiwan dragon kiln is streamlined, with thin ends, a large middle, a steep lower section, and a gentle tail section. The shape of the inner kiln cavity is smooth and reasonable, which is conducive to the rise and fall of hot gas and the control of atmosphere, and has excellent charging and firing performance [27] (Figure 6). At present, the largest dragon kiln in Shiwan is Nanfeng Ancient Kiln, which has been burning for 500 years [28].

### 2.2. Research Methods

Compared with the previous studies mostly based on the existing theoretical framework [29] or through the analysis and induction of collected data [30] to explain a certain cultural ecology, the paper attempts to explore the cultural ecology formed by the core of Shiwan pottery sculpture technique through a more scientific and standardized form, and selects the grounded theory as the method of this study. Grounded theory does not presuppose opinions, but discovers theories in the continuous comparison of texts, so it can better break through the limitations of the aforementioned study. It should be noted that this method involves the whole process from question posing, data collection, data processing, to theory construction. Grounded theory was first proposed by Glaser and Strauss [31]. It is different from the previous method of hypothesis testing and ethnography to handle materials, and can help people to create new theory from scratch. Later, due to different academic views, the grounded theory gradually diverged and developed into three different versions. The first is the classical grounded theory confirmed by Glaser’s book “Theoretical Sensitivity” [32], the second is the proceduralized grounded theory proposed by Strauss to standardize the grounded process [33], and the third is the constructivist grounded theory formed by Charmaz combining the theoretical perspectives of both and integrating his own new ideas [34]. The last version believes that the researcher constructs his own theory through participation and interaction with people, different perspectives, and research practices. Although there are still cognitive differences in the application of grounded theory, people can never ignore the great value of grounded theory for academic research.

This paper follows the core issue of “continuous comparison” in grounded theory. Considering the actual situation of research comprehensively, since the purpose of this study is to explore the evolution of the cultural ecology of Shiwan pottery sculpture technique, the process cannot be separated from human behavior to a large extent, and they will construct meaning for their own behavior. Therefore, the paper chooses the method of constructivist grounded theory. Subsequently, it identifies the following three main questions through the purpose: (1) What is the formation path of the cultural ecology of Shiwan pottery sculpture technique? (2) How is the Shiwan pottery sculpture technique inherited and innovated? (3) What is the concrete expression and significance of the new cultural ecology eventually formed by Shiwan pottery sculpture technique? Finally, the paper collects data according to this way, and conducts initial coding, aggregation coding and theoretical coding on valid texts to answer the above questions.

### 2.3. Data Collection

The data of qualitative research are texts, including both elicited texts and extant texts. To avoid the researcher’s preconceptions as much as possible, this study mainly focuses on extant texts, collects relevant data from multiple channels using online field surveys, and with the help of octopus collector—an intelligent automatic, multi-language free web crawler software (uses the built-in Python code), supplements by manual retrieval to ensure the accuracy and authority of data.

Selection criteria: Based on the principle of question-oriented and data-driven, the information selection criteria in this paper include: (1) The limitation of time. In 2007, Foshan carried out rectification and improvement for the ceramic industry, which became an important turning point [10]. In order to preserve the situation of Foshan ceramics before the transformation for comparison, this paper expands the range from 2000 to the present. (2) Ambiguity of search terms. Since this study does not carry out theoretical presupposition, only the five words “Shiwan”, “Shiwan pottery sculpture”, “Shiwan pottery sculpture technique”, “Shiwan Ceramic”, and “Foshan Ceramic” are used as the terms in the search; this approach is to ensure the diversity of text topics, to discover as many dimensions as possible in the subsequent phase. (3) Diversity and representativeness of data acquisition platforms. In order to ensure the coverage of effective information in the data to a greater extent, the data types selected in this study are diverse, covering information, news, official documents, papers, and books, considering both official and unofficial sources. In the platform selection, according to the factors such as platform positioning, popularity, number of users, activity and so on, several most-representative platforms of different types were selected, including Baidu, WeChat, Weibo, Superstar E-books, CNKI, Wanfang Service Platform, Duxiu Base, and the official government platforms that publish information related to Shiwan.

Retrieval process: (1) From 13 October 2022 to 21 October 2022, with the help of Python technology, the researchers used the aforementioned keywords to retrieve text, image, video, and other content generated by the three platforms of Baidu, Microblog, and WeChat, and used manual repeated data cleaning. A total of 2 rounds were carried out, and finally 399 reference materials were selected from the original 2140 materials. (2) From 21 October 2022 to 24 October 2022, the most commonly used, authoritative, and widely covered literature databases in China, including CNKI, Wanfang Service Platform, Duxiu Base, and Superstar E-book Platform were selected to search for the aforementioned keywords in the form of manual screening, downloaded papers and books related to the purpose of this research, and conducted a second search for papers in this process. (3) From 24 October 2022 to 27 October 2022, relevant policy texts were sorted out on different official websites in chronological order through manual screening. The final data sources and descriptions are as follows (Table 1):

Finally, the authors extracted the materials and saved them into Microsoft software. Among them, the MicroBlog sources were stored in the “. xlsx” format, papers and books were stored in the “. pdf” foramt, and other files were stored in the “.docx” format.

### 2.4. Data Processing

#### 2.4.1. Initial Coding

Initial coding is the process of defining the data content first, this stage will classify, summarize, and describe each part of the data. In the initial coding stage, the authors assigned a different letter to each piece of original data according to the data type, including message (A), information (B), paper (C), book (D), and official document (E), and used the sentence-by-sentence coding format without presupposition. In this way, the statement can be as close as possible to the original event, retain the original voice and tense, and discard personal subjective ideas, while avoiding too broad words in the coding process. At this stage, 1153 initial codes were obtained. Subsequently, the authors summarized, analyzed, and compared the different initial codes, further refined and combined the relatively concentrated content, and finally obtained 203 optimized codes as shown in Table 2.

#### 2.4.2. Focused Coding 

Focused coding, as the second major stage of coding, is more directional, selective, and conceptual than initial coding, and is used to synthesize and interpret a larger range of data. Through the cyclic comparison between data and initial code and between each initial code, the study comprehensively considered the year, action execution tense, character voice and other factors, to form the code that best reflects the essence of the data. At this stage, 49 aggregation codes and 21 categories were obtained, and different stages were preliminarily divided. Some examples are as follows (Table 3):

#### 2.4.3. Theoretical Coding

The theoretical coding process aims to explore the possible relationships between categories and specifying them, thus theorizing the stories. As an integrated framework, theoretical coding helps to clarify and enhance the researcher’s analysis. During this stage, the authors went through many explorations, constantly returned to the previous stage to ask questions, and corrected some of the aforementioned codes according to the original materials and supplementary theoretical literature [35,36]. Finally, the research formed eight theoretical codes, and sorted out the cultural and ecological evolution model of Shiwan pottery sculpture technique, as shown in Table 4.

#### 2.4.4. Theoretical Saturation Examine

**Figure 7:**
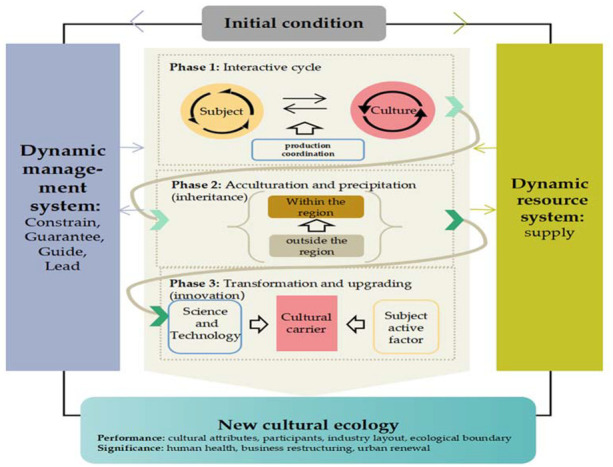
Cultural ecological evolution model of Shiwan pottery sculpture technique.

To more rigorously test the theoretical saturation of the present model, this study combined the researcher ensemble method with the analytical data ensemble method for testing, and established the detailed implementation principles for this. The details are as follows: (1) Set up an exclusive data retrieval team with four authors, one of whom supervised and guided the whole process as an industrial design guidance expert, and one of the other three used Python to analyze data for crawling, and two used manual methods in charge of data retrieval and subsequent data processing. (2) Two processors coded the same data, disassembled the grounded process and subdivided the steps, arranged the time planning reasonably, and ensured that both parties were at the same working pace as much as possible. (3) During the coding process, each author needed to continuously and repeatedly check the textual material, and timely discussed and changed after completing each step. For the textual material with inconsistent discussions, after excluding theories and cognitions with obvious errors, if there were still different opinions, they discussed with the supervisor in the working group and finally reached an agreement. (4) Considering that the characteristics of different software operations can make the research more convenient, Excel and Nvivo12 software were comprehensively used in the data analysis process. The use of Excel facilitated cleaning and adjustment of the data at any time, and after the two opinions were unified, it was imported into the Nvivo for the category’s coding. After the coding was completed, two authors rechecked and supplemented the contents of nodes and subsections under the Nvivo, and merged and reorganized some nodes to form a consistent coding node as much as possible. In the end, the work team reached an agreement, and no new concepts emerged during the coding process, which was considered to have passed the theoretical saturation verification [34], and the model was constructed as follows (Figure 7). More details were given in the “Section 3”.

## 3. Results

### 3.1. Initial Condition and Systematically Operation

The reform of the Shiwan pottery sculpture technique is formed under the profound historical background, and there is a big difference with the time node of 2007 as the dividing line. After long years of development, Shiwan pottery sculpture technique has accumulated a large resource base. With the advantages of natural environment and the support of national policies, Foshan ceramics has shown a continuous rapid development since the reform and opening up, accumulated a solid industrial foundation, a complete industrial system, diversified talents, etc., that has become “the largest building ceramic production base, ceramic chemical color glaze production base, ceramic commodity distribution and exhibition center in China” at one stroke (E-09). Before 2007, Foshan’s ceramic products were “mainly architectural ceramics and sanitary ceramics, as well as special ceramics, art ceramics, garden ceramics, and a small amount of daily ceramics and refractory ceramics” (E-11) [10]. The inheritance of Shiwan pottery sculpture technique has also formed three kinds of stable master–apprentice systems; family inheritance and further study, as well as the guilds around the country have fixed rules of the trade. However, this extensive economic development model quickly exposed obvious disadvantages with the progress of the times. At the end of the 20th century, the production model of daily ceramics caused serious environmental pollution in the local area, threatening people’s health [10], and coupled with the complex and changeable international trade environment and the modern development trend of the city, the survival space of Shiwan pottery sculpture technique was squeezed, and the city’s economic and social development faced great pressure and challenges. On the other hand, under the original cultural system, the cultural inheritance is basically limited to internal personnel, and this system has led to the aging of the inheritance subject, the lack of academic theory, and the inconsistency of evaluation standards for Shiwan pottery sculpture techniques in different regions, limiting its further development. The above predicament directly and continuously affects the daily experience of residents, thus giving rise to a strong demand for energy conservation and emission reduction, landscaping, industrial transformation, and product promotion. Under the joint action of these elements, the original ecology urgently needs to be adjusted, thus driving a new round of cultural ecology evolution.

When the dynamic balance of the original cultural ecology is defeated, the original elements will flow in the form of resources and information, forming management and resource systems. Specifically, the resources within the resource system include natural resources such as geographical location and climate conditions, as well as social resources such as talents, site space, and market reputation. The resource system plays a supply role in the subsequent phase, while results generated will be fed back to the resource system to keep it always in a dynamic state. In the same way, information is regarded as the product of human processing, which mainly forms a management system. The management system also has an effect on the process of each phase and receives feedback. Through continuous improvement, the management system forms a modern social governance system from the state to local governments and then to the grassroots. With the guarantee means of law, directory establishment and financial support, with the guiding ideology of inheritance purpose, academic research and urban construction planning path, and by taking the lead in establishing a number of urban benchmark projects to drive the development of other industries, it mainly provides regulation, guarantee, guidance, and leadership for the evolution phase. Since the interaction between the two systems and phases is always continuous, the subsequent phases will not be repeatedly emphasized under unnecessary conditions.

### 3.2. Phase 1: Interactive Cycle

When the original cultural ecology of Shiwan pottery sculpture technique changed, the people living in this region first played a key role. Craftsmen with the mission of inheriting the Shiwan pottery sculpture technique and entrepreneurs with the mission of saving the Shiwan ceramic industry are regarded as core figures. They have noble characters and tenacious spirits, consciously seek change, innovate with determination, and walk at the forefront of the times. Many craftsmen successively created new pottery concepts, continuously promoted intergenerational inheritance, and attracted young people to engage in pottery work. For example, “Liu Chuan, an art master, is the first person to systematically summarize centuries-old Shiwan pottery sculpture theory” (A-71), “Chen Yuehua, a female character, takes office in danger and wins a turning point for the survival of the state-owned Mei pottery factory" (A-343). In addition to the core characters, the support of other characters also plays a vital role, they have different classes, identities and ages, and play their own unique roles. Young pottery creators who grow up in the new generation often have more advanced visions and trendy ideas. Based on absorbing and inheriting the technique and charm of Shiwan pottery, they constantly integrate innovative modern aesthetic elements, and can “integrate science fiction and animation elements into Shiwan pottery sculpture creation”(B-22), “walk between inheritance and innovation by means of cultural and creative design” (A-247), “dare to infuse traditional pottery into contemporary life aesthetics and create Shiwan pottery space” (B-16) College students actively use their professional knowledge through social practice, or investigate and put forward opinions on Shiwan pottery sculpture skills, or use new media to help the cultural dissemination of Shiwan ceramic sculpture skills. The pottery cultural elites put forward suggestions for cultural development through political participation. As direct cultural beneficiaries, common people can also give feedback through personal experience. The differentiated and diversified demands promote and lead the production direction of the ceramic products and cultural renewal. Because “the inheritance of intangible cultural heritage needs to involve common people and share the achievements of cultural heritage” (A-23), “Shiwan ceramic needs to make systematic changes according to market demand” (A-76). In terms of social organizations, including the media, industry associations, social welfare organizations, and so on also play an important role. For example, associations promote education and academic activities, and the media mainly help cultural dissemination. 

In addition to the role of various types of principals, characters and organizations do not exist in isolation. They interact with each other and finally produce positive or negative results. For example, cooperative promotion “cooperating with the media is the promotion path for original works adopted by the younger generation of pottery artists” (A-26), refeeding growth “collectors can further refeed the Shiwan pottery industry and provide effective guidance for the creative direction of artists” (A-79), and leading demonstration “Liao pottery studio in Shiwan has become a national level living heritage model studio for intangible cultural heritage inheritors” (A-451) are all positive effects generated through the joint subject. At the same time, due to the differences of different groups, people cannot avoid conflicts of interest in the process of association. For example, there is a factional struggle between the traditional faction and the modern faction in Shiwan pottery sculpture technique (A-250). For the Shiwan pottery sculpture technique that has a long history, it has a strong cultural inclusiveness. It can not only integrate traditional culture such as calligraphy, painting, and paper-cutting, but also “fit modern aesthetics and cater to trendy consumption concepts” (C-32) [37], interacting with other cultures in the region. Moreover, it also interacts with people, and human beings realize their own demands through the use of culture. Culture plays the roles of education and ideological cultivation, which improves the quality of life. In this interactive process, it can help culture to reproduce, for example, the introduction and promotion of culture further expands the cultural ecological area of Shiwan pottery sculpture technique.

During the interactive cycle stage, there are interactions among people, cultures, and human beings. These interactions occur at the same time, and the result of a certain process constitutes a new condition, thus the interaction is cyclical. In addition, the interaction between subject and culture is also coordinated by production aspects such as the balance between art and industrialization, and the benefit ratio between quality and output, showing the result of either confrontation or promotion. 

### 3.3. Phase 2: Enculturatioin and Precipitation 

As the results of the previous phase are exported, they become the conditions for the next phase when a certain limit is reached. Due to the interactive cycle phase in which humans promote the culture of the Shiwan pottery sculpture technique and introduce external regional culture, these results make the Shiwan pottery sculpture technique in fact influenced by external ecosystems and foreign cultures. The promotion of culture leads to the expansion of cultural ecological boundaries. Although the specific information of foreign cultures also enter the region when they are introduced, this process cannot be observed and measured, and the scope of occurrence still remains within the region; so this study defines the boundary between phase 1 and phase 2 as the beginning of the event of cultural–ecological region expansion.

Specifically, the regional promotion of the Shiwan pottery sculpture technique by humans is reflected in different regions and countries in China. These cross-regional communication activities have promoted the popularity of Shiwan pottery sculpture technique, brought more discussions and excellent experience, and finally produced a new situation of cultural adaptation. For example, “The Shiwan pottery sculpture works of teachers and students in Dongping Primary School have repeatedly won awards and have gone overseas, becoming the bridge to spread and introduce Chinese Shiwan pottery art” (A-251), “Foshan and overseas build a business relationship of ’manufacturing and commercial trade’” (A-264), “Shiwan Art Pottery are highly praised by guests from China and Germany” (B-13), “Macao art museum exhibits Shiwan ceramics exhibition” (B-34), “Shiwan organizes a study group to learn from experiences at home and abroad” (A-85), etc. These results in phase 1 are regarded as phase 2 conditions. As it is difficult to directly observe the influence of Foshan on other areas, this study mainly focuses on the core area of the cultural ecology of Shiwan pottery and plastic technology, namely Foshan. The behavioral situation in this phase is mainly regarded as the effect of various elements outside the region on the cultural results of the Foshan region, which can be understood as the process of cultural acculturation. As an answer to how the two cultural groups are “acculturating”, the acculturation strategy mainly answers two questions: “What extent does the original culture remain?” and “What extent does the contact and participation in the new culture change?” [38]. For the Shiwan pottery sculpture technique, cultural acculturation shows a relatively positive process, such as exchange, cooperation, reference, etc. Therefore, the preservation and change of the original culture is a positive direction of “taking the essence and discarding the dross”. The result of this process will continue to precipitate. This precipitation is not simply overlap and duplication, but through this process, the Shiwan pottery sculpture technique realizes the innovative inheritance of culture. 

### 3.4. Phase 3: Transformation and Upgrading

When the results of phase 2 accumulate to a certain extent, there will be a transformation from quantitative change to qualitative change, and innovation will be realized in this process. At this phase, the role of science and technology and subjective initiative is more prominent. Science and technology have innovation on the industrial and digital levels. The result of this effect is breakthrough, making the industry more civilized, such as “Ceramic industry develops new technology and environmental protection projects, environmental protection technology and equipment, supports technical transformation, and promotes the application of antipollution and emission reduction technology” (E-33), “Complete the data collection of Shiwan ceramic works with 3D digital acquisition technology” (C-04) [39] are all important embodiments. The subject active factor is mainly manifested in the creative design. The process is to “highlight local humanities with creativity, organically link people with local culture, and integrate intangible cultural heritage into modern life” (A-46), “link culture and industry with design, allowing pottery and building pottery enterprises to explore the integrated development path of ’design, culture, industry, tourism, art and creativity’”, and promote Foshan to become the design highland in China’s Greater Bay Area” (E-66). Compared with the former, its effect on culture is a gradual approach. The adjustment of the two with the function of the dual systems finally produces innovative results and promotes the emergence of a new cultural ecology. 

The new cultural ecology is more active and healthier, which is culture-oriented and aims to build a healthy ecological city with the ultimate goal of realizing human well-being. The content of the new cultural ecology is expressed in four dimensions: (1) Cultural attributes. Cultural attributes include the inherent attributes of culture such as shape, material, process, etc., as well as the functional attributes of culture on human beings. The innovation of cultural attributes, on the one hand, is reflected in the creators’ adaptation and integration of the thinking innovation and material innovation proposed by the times, on the other hand, it is also reflected in the great satisfaction of culture to human needs. For example, culture benefits human life, “the construction of the artist village ecosystem has improved the cheap ceramic tiles on the surface of the ancient stove kiln base and the disorderly residential areas” (A-481), and “ceramic enterprises try to closely combine space and art, and convey to people the popular trend of design leading future life” (A-298). (2) Participants. With the joint efforts of groups mainly composed of core figures and social organizations, more and more people pay attention to and devote themselves to the Shiwan pottery sculpture technique, and the participants are diversified and younger. (3) Business layout. Nowadays, the Shiwan ceramic industry has shown a development trend of intelligence, environmental protection, and high-end, and the added value of culture has also been increasing, leading to the creation of a number of new industrial forms and a more coordinated layout of business types. (4) Ecological boundary. Due to the cross-regional flow and interaction of culture, the boundary of cultural ecology has been further expanded. 

In a practical sense, due to the industrial improvement of the Shiwan pottery sculpture technique, such as the use of more environmentally friendly materials and smarter auxiliary equipment, the harm of ceramics to human health has been greatly reduced. At the same time, more ceramic works with cultural and artistic attributes have appeared in the industrial restructuring, and the cultural attributes of Shiwan pottery sculpture techniques have been further strengthened, and the social atmosphere has become more profound. Especially in terms of education, many young pottery lovers have been trained through theme lectures, cultural activities, and other ways. The research program of inheritors of intangible cultural heritage helps craftsmen adapt to the new changes of the times. In terms of urban construction, Foshan put forward the slogan of establishing a culturally-oriented famous city and took Shiwan pottery sculpture technique as an important object. During the construction process, many old cities have been transformed and have assumed the role of talent gathering, industry gathering, and professional demonstration. At the same time, in recent years, Foshan’s urban environmental art has begun to open to the use of local profound pottery cultural resources. These ceramic public arts have formed a new cultural landscape, such as “Shiwan Park uses 365 vats of different colors made by Shiwan pottery sculpture technique to recreate art in the form of installation art” (C-25) [11] which not only creates an atmosphere of innovative culture, but also shows regional inclusiveness. Therefore, pottery culture plays an important role in reshaping and enhancing Foshan’s personalized city image.

## 4. Discussion

Although the discussion of cultural ecology has been applied to many fields, there is still a phenomenon of emphasizing local discussion and neglecting theoretical construction to a certain extent. For example, when discussing a certain cultural ecology of a certain region, many researchers often focus on the development of the culture itself, without comprehensively considering the various elements from a systematic perspective. Some common research fields include cultural values such as belonging [40], physical and mental health [41], and cultural practices such as subject identity construction [42] and art education [43], or put forward some targeted solutions from the dilemma of culture [44,45,46]. There are also studies that use the method of text induction [30] or case comparison [47] to integrate and elaborate the acquired materials. In contrast, this paper takes a higher perspective, linking cultural inheritance and innovation to urban development and human well-being, pointing out that cultural innovation can have a “purification” role in ecology. At the same time, this study introduces the method of grounded theory, taking into account the discussion of time and space dimensions, so the presented theory not only reveals the process of interaction between various elements and the way of cultural inheritance and innovation in this process, but also reveals the results of the new cultural ecology in detail. The use of Python technology also makes the data collection process more standardized. 

Besides, ecosystem services are a premise and guarantee for satisfying the multi-levels of human well-being [2], and existing research usually divides ecosystem services into four categories: supply, regulation, support, and cultural services, among which cultural or landscape services are usually considered to have a positive effect on improving human physical or mental health [48]. However, there is also a scholar who pointed out that some supply services also have strong cultural importance [49]. Therefore, this paper explores the various functions of cultural ecology by constructing a cultural ecological evolution model of the Shiwan pottery sculpture technique, and points out that culture should not only be beneficial to people’s physical and mental health, but also be regarded as a sustainable resource, which can drive urban development through a series of action mechanisms and create a more harmonious social atmosphere. Taking Shiwan as an example, the culture of pottery sculpture technique has played many roles in linking regional cooperation, feeding back industrial development, and improving the city’s image. These values far exceed the pleasure function defined by cultural services in the existing ecosystem.

Finally, this study explores from cultural ecology theoretical perspective, the collected and processed data covering the comprehensive information on the Shiwan pottery sculpture technique since 2000, the content of the text is rich and detailed, and the final theoretical model can serve as a reference for the future development of the Foshan ceramics industry to a certain extent, enriching the theoretical research of the Shiwan pottery sculpture technique. As a city from manufacturing to cultural industry, Foshan has created a sustainable development path through the inheritance and innovation of Shiwan pottery sculpture technique, which can provide an example for the development of other similar cities.

## 5. Conclusions

In the background of accelerating urbanization, this paper focuses on the physiological health of people and urban renewal under environmental changes, aiming to explore how to achieve human well-being by establishing a sustainable ecosystem model in urban space. Therefore, this paper chooses Foshan as the case study. This city has both the titles of a famous historical and cultural city and an industrial manufacturing city. The ceramic industry is one of the pillar industries in Foshan. With the development of society, the ceramic industry inevitably brings serious environmental pollution. This situation has not been gradually improved until the beginning of the 21st century. Eventually, Foshan realized the transformation of the city and moved towards a healthier development path. Against this background, this research constructs the cultural ecological evolution model of Shiwan pottery sculpture technique, explores how was Shiwan pottery sculpture technique inherited and innovated, and explores and clarifies the path and significance of the role of many elements in the ecosystem. As the core of ceramic production, the Shiwan pottery sculpture technique is not only a craft technology, but also the first batch of national intangible cultural heritage in China. The dual attributes of industry and culture make Shiwan pottery sculpture technique widely link various industrial chains in life and production, and the inheritance and innovation of Shiwan pottery and plastic technology promote social progress. For example, Foshan has continuously improved its industrial technology to make it environmentally friendly, high-end, and intelligent. Moreover, relying on the ability of Shiwan pottery and sculpture technology, the Foshan government has created a series of cultural landscapes and a number of emerging industrial forms, which also bring new beauty to the city and the people living in it.

From the perspective of cultural ecology, this paper uses grounded theory and crawler technology to obtain a large amount of public data on the Internet. After repeated sorting and induction, about 860,000 Chinese texts are finally determined as the source of this coding data. Then, the initial coding, aggregation coding, and theoretical coding are used to go deeper and deeper, and the model of this coding is finally constructed through the continuous comparison and theoretical saturation verification.

Finally, this study explains the specific operation mode and significance to provide reference for the transformation of other industrialized cities. In order to ensure that the study can explore many potential elements as widely as possible, in this paper, a large number of existing texts are selected as the original material for this study. The partial imbalance of data content proportion is one of the insufficient studies in this paper. In addition, even though the implementation steps of the detailed rules have been determined in the grounded theory process to prevent authors from producing subjective biases as much as possible, it still cannot be completely avoided in the actual operation process, and there is an overall lack of verification of the current theoretical model from the perspective of quantitative analysis. In short, building on this study, the next step of research can consider digging deeper into an association, developing individual interviews and expanding the object, while revising the research process, gathering more authors to participate in coding, and using multiple methods for complementation, in order to adjust the subsequent model.

## Figures and Tables

**Table 1:** The data source and effective quantity description of Shiwan pottery sculpture technique since 2000.

Data Type	Source Platform	Platform Description	Valid Quantity	Valid Text Quantity
Message	Baidu, WeChat	Baidu: the world’s largest Chinese search engine WeChat: mainstream instant messaging software in China	295 entries	489,291
Information	MicroBlog	A domestic mainstream social media platform that shares and disseminates information based on user relationships, and shares brief real-time information through the attention mechanism	104 entries	22,767
Official Document	Guangdong Administration Service Website, Foshan Government Website, Foshan Daily Digital Newspaper Website, Chancheng Government Website, Shiwan Subdistrict Office Website, Foshan Ceramic Industry Association Website	Official platforms to release relevant information about Shiwan, Foshan, Guangdong	18 entries	69,458
Book	Superstar E-books	One of the largest Chinese online libraries, with more than several millions of Chinese books, covering various disciplines	4 copies	203,122
Paper	CNKI, Wanfang Platform, Duxiu Base	The most authoritative and widely used database of books and documents in China with the widest coverage of material information	19 articles	82,154

Note: Judging whether it is a valid text, mainly follows the time limitation and content relevance. The counting unit of the valid text here is Chinese characters.

**Table 2:** Initial coding analysis results of Shiwan pottery sculpture technique’s effective text (some examples).

Optimized Code	Initial Code	Representative Original Material
Platform construction	Develop product communication platform	A-14 Develop a communication platform for ceramic creative products
Industry transformation	Manufacturing turns to cultural business and industries	A-42 Pottery sculpture technique changes from manufacturing to cultural business and industry
Design empowerment	Industrial design improves quality	A-101 Industrial design is an important part of improving ceramic quality
Property protection	Accelerated copyright registration	E-20 Shiwan pottery sculpture technique accelerates for copyright registration
Practical experience	Experience the culture personally	B-04 Experience Shiwan pottery sculpture technique personally
Responsibility	Inheritors take responsibility consciously	C-18 Inheritors consciously take responsibility to promote cultural inheritance and innovation
Associated opportunity	Interest motivates	A-67 Interest drives creator to make pottery a lifetime career
Master-Apprentice heritage	Adopting new apprentices to inherit	B-21 Huang Zhiwei accepts new apprentices to inherit pottery sculpture technique
Status evaluation upgrade	Shiwan products won international acclaim	B-13 Shiwan Art Pottery are highly praised by guests from China and Germany
Standardize industry construction	Improve pollution production process of enterprises	E-16 Renovate and control the polluting production process of enterprises
Study development experience	Organize to learn from other places	A-85 Shiwan organizes different delegations to learn from experience at home and abroad
Inconsistent standards for value assessment	Differences in Aesthetic Cognition between North and South	D-21 Due to the differences in aesthetic cognition between the north and south, there are some misunderstandings about Shiwan pottery sculpture in the northern market

**Table 3:** Focused coding analysis results of Shiwan pottery sculpture technique’s effective text (some examples).

Category	Aggregation Code	Optimized Code
Basic elements	Original environmental base: resources, information	Original natural resources, social environment, industry system, academic foundation, industry advantages, and artistic atmosphere
	Traditional inheritance method	Family inheritance, craftsman inheritance, further study, and master-apprentice inheritance
	Inherent cultural attribute	Cultural connotation, cultural characteristics, artistic features, reflection of the times, cultural value, and creation rooted in the times
	Cultural functional attribute	Meet different needs, improve human life quality and academic value
Adjustment elements	Coordinate production	Coordination between industrialization and artistry, artistry and daily life, tradition and innovation, quantity and quality, function and aesthetics……
Innovation elements	Science and technology promote innovation	Digital transformation, digital technology enhanced experience, application of digital technology storage, digital technology empowers dissemination, creation and publication of digital art works, technology empowers development, industrial technology innovation
	Main active factors promote innovation	Creative link, design empowerment
Subject characteristics	Subject consciousness	Self-awareness in responsibility and thinking
	Subject leading	Case of living inheritance model studio for intangible cultural heritage inheritors……
Industry layout innovation	Industrial reform and development	Industrial intelligence development, industrial environmental protection development, industrial high-end development
	Industrial planning execution	Layout planning, industrial transformation, industrial gathering, location positioning, industry planning, industrial goals, industrial transfer
	Industry ecological reconstruction	Industry association, industry transformation, industry planning
New cultural ecological Significance	Effect on people	Culture benefits human life, culture leads future life trends
	Effect on industry	Culture promotes economic development, culture feeds back industrial development
	Effect on the city	Pottery sculpture technique enhances city image, culture integrates into urban space

**Table 4:** Theoretical coding analysis results of Shiwan pottery sculpture technique’s effective text (all examples).

Theory Code	Category
Initial conditions	Basic elements, variable elements
Dynamic management system	Governance system, guarantee means, guiding ideology, leading specimens
Dynamic resource system	Resource supply
Interactive cycle	Intercultural interaction, culture-human roles, interhuman interaction, human-cultural roles, regulatory elements, subject characteristics
Transformational change	Innovation elements
System information and resource flow	Environment acts on culture, environment acts on humans, humans react on environment
New cultural ecological performance	Cultural attribute innovation, business layout innovation, inheritance subject innovation
New cultural ecological Significance	Ecological significance of new culture: human health, business restructuring, urban renewal

## Data Availability

All data were derived from resources available in the public domain. Due to the large sources of data, readers can contact the corresponding author to provide all the original materials if necessary.
